# Applications of GIS and geospatial analyses in COVID-19 research: A systematic review

**DOI:** 10.12688/f1000research.27544.1

**Published:** 2020-11-27

**Authors:** Rakibul Ahasan, Md. Shaharier Alam, Torit Chakraborty, Md. Mahbub Hossain

**Affiliations:** 1 Nature Study Society of Bangladesh, Khulna Unit, Khulna, 9000, Bangladesh; 2 EviSyn Health, Khulna, 9000, Bangladesh; 3 Texas A&M University, College Station, Texas, 77843, USA; 4 Asian Disaster Preparedness Center, Rajshahi, 6202, Bangladesh

**Keywords:** GIS, Coronavirus, COVID-19, Spatial analysis, Systematic review, Evidence-based practice

## Abstract

**Background:** Geographic information science (GIS) has established itself as a distinct domain and incredibly useful whenever the research is related to geography, space, and other spatio-temporal dimensions. However, the scientific landscape on the integration of GIS in COVID-related studies is largely unknown. In this systematic review, we assessed the current evidence on the implementation of GIS and other geospatial tools in the COVID-19 pandemic.

**Methods:** We systematically retrieved and reviewed 79 research articles that either directly used GIS or other geospatial tools as part of their analysis. We grouped the identified papers under six broader thematic groups based on the objectives and research questions of the study- environmental, socio-economic, and cultural, public health, spatial transmission, computer-aided modeling, and data mining.

**Results:** The interdisciplinary nature of how geographic and spatial analysis was used in COVID-19 research was notable among the reviewed papers. Although GIS has substantial potential in planning to slow down the spread, surveillance, contact tracing, and identify the trends and hotspots of breakdowns, it was not employed as much as it could have been. This review not only provided an overarching view on how GIS has been used in COVID-19 research so far but also concluded that this geospatial analysis and technologies could be used in future public health emergencies along with statistical and other socio-economic modeling techniques. Our systematic review also provides how both scientific communities and policymakers could leverage GIS to extract useful information to make an informed decision in the future.

**Conclusions:  **Despite the limited applications of GIS in identifying the nature and spatio-temporal pattern of this raging pandemic, there are opportunities to utilize these techniques in handling the pandemic. The use of spatial analysis and GIS could significantly improve how we understand the pandemic as well as address the underserviced demographic groups and communities.

## Introduction

COVID-19 has taken the world within a blink of an eye with a rapidly increasing confirmed cases and case-fatalities around the world ^1^. After originating in the Hubei province of China in late December 2019, the World Health Organization (WHO) termed it an epidemic on January 29 2020 ^2^, named it COVID-19 on February 11, and declared it as a pandemic on March 11 ^3^. Although the first reported case was in China, and it was the epicenter of the pandemic, the virus has mutated and changed transmission pattern several times since then. Lately, the United States, parts of Europe, and countries in the Global South had been reporting the highest number of cases with a rapid increase in both confirmed cases and fatalities ^1^.

The declaration of COVID-19 as a pandemic and subsequent lockdown at various levels, from the local city level to the country level, has a much broader impact on our surrounding environment compared to what we usually observe. Despite the availability of data, studies examining the impacts of this ongoing pandemic and enforced lockdowns using different geospatial analysis techniques is not substantial. However, the application of spatial analysis tools, techniques, and geographic information system (GIS) platforms provide the scientific community and the practitioners a wide range of benefits. These benefits include more straightforward and understandable visualization, real-time tracking of confirmed and reported case numbers ^4^, contact tracing, spread direction, and also, to identify the hotspots to limit the dispersion and community spread ^5, 6^. The application of GIS in public-health related issues is not something introduced during this pandemic. It was used by numerous studies in the past, long before the computerized GIS software was born in the mid-1960s ^7^. Since then, GIS was extensively used in analyzing, visualizing, and detecting patterns of disease. A recent review found that among the included 869 studies, one-fourth of the studies used GIS techniques for mapping, especially infectious disease mapping ^8^.

Different GIS software and methods have been implemented and widely accepted to prevent the transmission by imposing lockdowns and contact tracing. The best example of GIS application during this pandemic is the web-based near-real-time COVID dashboard created by the Johns Hopkins University ^4, 9, 10^. Later the WHO and different local and regional governing bodies also followed the same direction ^11^. The online dashboards have been a critical source of information during this pandemic. Although at the beginning the studies implementing or using GIS methods were more focused on visualizing or contact tracing, later, they moved on spatial analysis incorporating social, economic, environmental, and more sophisticated analytical tools as more data started to become available.

There have been attempts to review the studies regarding the application of geospatial analysis in COVID-19 related studies. Pardo *et al*. (2020) reviewed the studies that were focused on understanding the distribution patterns of the pandemic and identified such applications in six thematic groups ^12^. Similar attempts were made by Kamel Boulous and Geraghty (2020) to review the web-based use of GIS technologies ^6^. However, none of these approaches followed a systematic approach in selecting the articles, and the reviews were mostly incomprehensive.

Systematic reviews provide an organized, replicable, and methodologically synthesized landscape of evidence that may inform policymaking and practice. During this pandemic, the scarcity of evidence remains a major challenge for public policymaking, which necessitates a careful assessment of the growing body of literature on GIS and geospatial analyses. Also, systematically evaluated evidence is critical for the advancement of science as further primary studies or research syntheses can be informed by the findings of a systematic review. We acknowledged this knowledge gap and conceptualized this review to advance science and practice related to GIS and a wide range of geospatial techniques that are being used in this pandemic. The objective of this study is to conduct a systematic review of the implementation of GIS and other geospatial tools and technologies in COVID-19 related studies. We highlighted the works that used geospatial techniques as part of their analytical method and tried to provide pointers on how these techniques can be better used in times of future public health emergencies.

## Methodology

### Guideline and data sources

This systematic review was conducted using the reporting guidelines as stated in the Preferred Reporting Items for Systematic Reviews and Meta-Analyses (PRISMA) statement ^13^. A protocol was prepared before conducting this review that was not registered with PROSPERO or any other organization. This protocol was uniformly followed by the reviewers at each stage of the review, which is available upon request. The data for this systematic review were retrieved from MEDLINE and Web of Science. Both databases have their own competitive advantages that provide a broader coverage of scholarly articles. MEDLINE is considered as the largest bibliographic databases for health sciences, whereas Web of Science provides access to journals from multiple scholarly disciplines. We used the following steps to identify relevant literature for this review. First, we used the following search query in each database: (“COVID-19” OR “2019-nCoV” OR “2019 coronavirus” OR “2019 novel coronavirus” OR “novel coronavirus” OR “SARS-CoV-2”) to identify COVID-19 related studies. Further, we used another search query to retrieve GIS-related studies as following: (“GIS” OR “ArcGIS” OR "Geographic information systems" OR "Geographic mapping" OR "Spatial analysis" OR "Geospatial analysis"). At the next step, we combined both these queries with “AND” operator to identify literature that is likely to contain studies referring to both these topics. Furthermore, as COVID-19-related literature is evolving rapidly, we also searched the Google Scholar database to identify studies that may align with the objective of this review. Also, we performed a reference searching and contacted subject matter experts for additional studies beyond the scope of the databases, if there were any. We limited the timeline for literature searching between 2019 and 2020 considering the origin of the outbreak in late 2019. The search was first conducted on May 7, 2020 and updated on June 28, 2020.

### Eligibility criteria

Articles were considered eligible for this review if they were a) published in English language, b) available as peer-reviewed journal articles, c) the primary focus of the paper was on any aspect of the COVID-19 pandemic, d) demonstrated the applications of GIS or geospatial analyses. Any article that did not comply any of these criteria were excluded from this review. For example, non-English studies, articles that were not peer-reviewed (e.g., letters, editorials, comments), studies that did not focus on COVID-19, or did not use GIS or geospatial techniques (conceptual papers without providing any findings) were considered ineligible for inclusion.

### Selection of studies and synthesis of evidence

All citations retrieved through database searching were imported in Endnote reference manager software for curating the collective bibliography. Further, this library was exported to Rayyan QCRI, a cloud-based software for citations screening and assessment. Two authors independently assessed each citation against the eligibility criteria stated above. At the end of the process, a third reviewer was consulted to review the conflicts and a consensus on inclusion or exclusion was made upon discussion. The full texts of the primarily selected articles were reviewed and reviewed by all authors.

Further, articles fulfilling all criteria for this review were retained and data were extracted using a predesigned extraction sheet in Microsoft Excel on the following variables:

a) publication details,

b) study objectives,

c) the sources of data,

d) countries of origin,

e) COVID-19 specific domain presented in the papers,

f) the use of GIS or geospatial analyses, and

g) the research outcomes or key findings of those studies.

The applications of GIS or geospatial analyses on COVID-19 studies were identified and narratively synthesized as major themes alongside tabulation of the key findings. A narrative synthesis is appropriate where quantitative or qualitative synthesis may not be feasible due to the methodological differences and other measures of heterogeneity across the included studies ^14^. Moreover, quality appraisal or risk of bias assessment were not considered in this review due to a large volume of studies and profound heterogeneity in methodological approaches, data sources, measurements, types of applications adopted in different contexts, and research outcomes in respective studies.

## Results

### Summary and characteristics of reviewed articles

**Figure 1.**
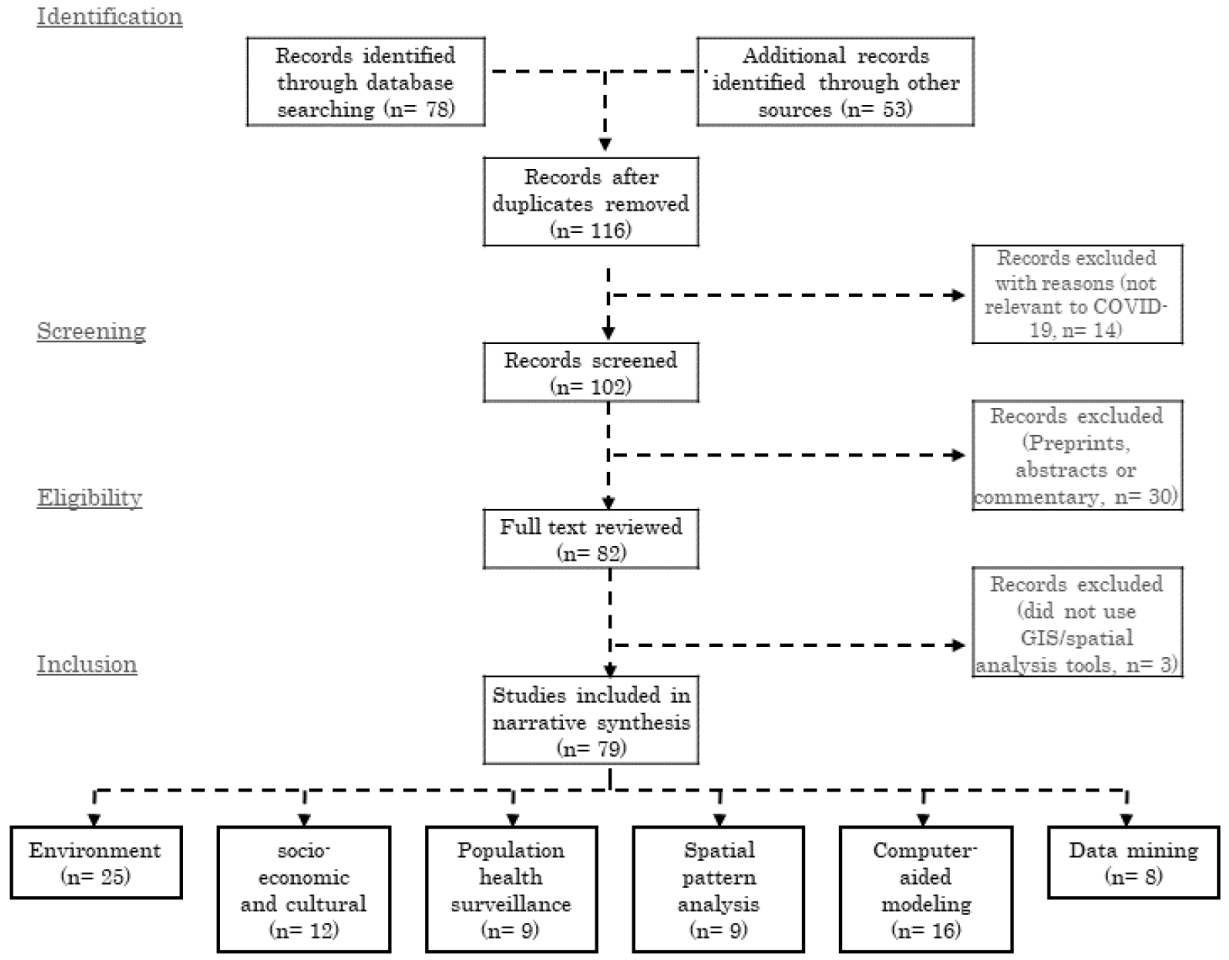
GIS Applications in COVID-19 research literature search and evaluation for inclusion.

**Figure 2.**
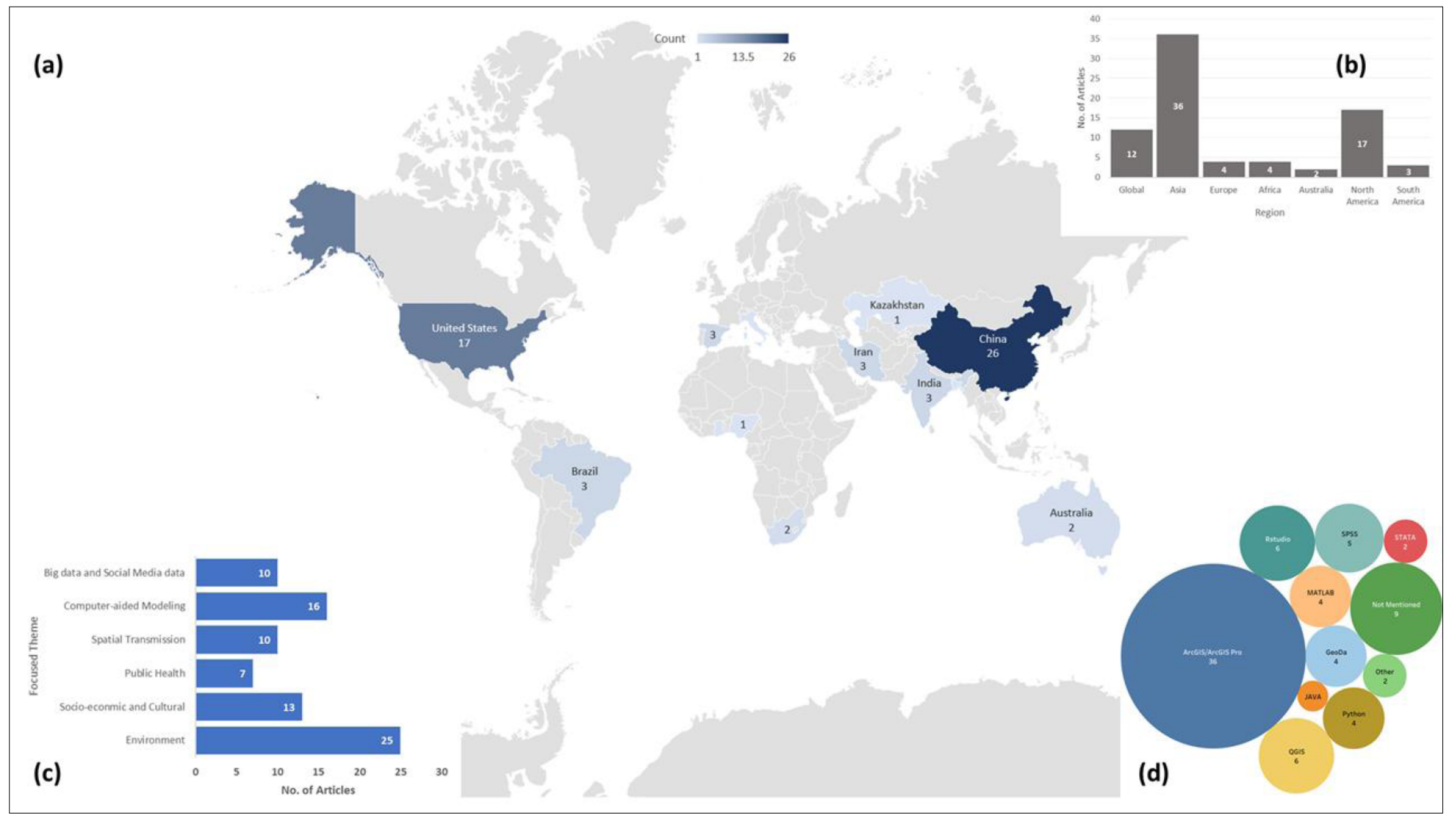
( **a**) article count per country and geographic distribution of the study sites; ( **b**) number of articles in each continents based on study site along with Global scale studies; ( **c**) number of articles in each thematic groups used in this review; and ( **d**) bubble chart showing the frequency of each software or tool used for spatial analysis.

The PRISMA diagram for this review is shown in Figure 1. We found a total of 79 articles which met all the inclusion criteria. A PRISMA checklist enlisting the contents of this systematic review is available on Open Science Framework repository ^15^. A summary of these 79 reviewed articles are listed in *Extended data*, Table 1 ^16^. The articles included in the review were published in a diverse group of journals, mostly in public health, urban planning, geography, and interdisciplinary journals. Although COVID-19 is more of a public health and welfare issue, these articles covered from public health issues to planning techniques, environmental concerns, and, most importantly, used geospatial analysis tools and techniques as part of their methods. In total, 32.9% of the articles (n=26) used China and 21.5% the United States (n=17) as their study site, which indicates to the disparity of the geographic coverage. Figure 2 shows the geographic distribution of the study area used in the 79 articles we included in our review. Wuhan, China, first reported COVID-19 cases, and the GIS has the highest number of confirmed cases, which also was reflected in the over-representation of these two countries in these articles. Africa and Europe had the lowest number of articles (n=4). Among these reviewed articles, 15% (n=12) worked on a global or multi-national scale. The global studies are more focused on mobility and how COVID-19 transmitted via airports and other human mobility ^17– 19^.

A wide range of geospatial tools were used in the reviewed articles. Most of these articles used regression analysis (n=18) and correlation analysis (n=11) for statistical analysis. Most prominently used spatial analysis was hotspot analysis using either kernel density function or other density techniques (n=16) followed by the spatial autocorrelation analysis using either global or local Moran’s Index (n=13) and proximity analysis (n=5). All the articles used different visualization techniques to display either the intermediate or the end products. Along with that, suitability analysis (n=3) and sensitivity analysis (n=4) was also used to find either access to hospitals or how the cases are distributed. One of the less frequently implemented but interesting technique was to track transmission patterns using the mobility data ^20– 22^. Similarly, remote sensing data was used for air quality and pollution measurement, but not that prominently (n=6). Nighttime imagery and solar radiation data were used by n=5 studies to compare the energy consumption differences between before and after pandemic situation.

ESRI ArcGIS is the most used platform (n=36) in these articles, but other open-source platforms like RStudio, where spatial and statistical analysis can be done simultaneously, were often used as well (n=6). Besides RStudio, QGIS (n=4) is another open source software platform that was highly employed by the researchers. Apart from that, a couple of the studies developed their own platforms and one of the studies used GPS. data. Most of these articles used COVID-19 confirmed cases as part of their analysis (n=77). In most cases, researchers used either WHO provided data or JHU provided data for the research purpose. As several spatial analysis was performed based on remotely sensed data, NASA and USGS satellite images were the primary source for those (n=5). Apart from that, the air quality index (AQI) was used in the articles focused on environmental analysis (n=8). As mentioned in the methodology, we did not assess the risk of bias among studies included in this review.

### Thematic groups

The rapid diffusion of COVID-19 and data convenience had enforced the global scientific community to work more vigorously on geospatial analysis of this pandemic. These studies had focused on distinct aspects of the pandemic and have different inputs. The articles included in this review was divided into six thematic groups- environment (n=25), socio-economic and cultural (n=13), Population Health Surveillance (n=8), spatial transmission (n=9), computer-aided spatial and statistical analysis and modeling (n=17) and big data, social media and mobile data (n=10).

***Environment***. We found a total of 25 articles that emphasized the use of GIS and spatial analysis on environmental issues related to COVID-19 ^18, 20– 43^. Although these studies had a similar research interest, they differ significantly based on the spatial scale- from global ^23– 27^ to regional ^28, 29^, national ^30– 38^ and small local scale studies ^39– 45^. Several works examined the relationship between different meteorological factors and the transmission potential of the virus. They included a diverse set of characteristics in the studies: temperature ^21, 25– 27, 30, 31, 33, 35– 37, 39, 42, 44, 46^, humidity ^21, 26, 27, 31, 33, 35– 37, 39, 44, 46^, precipitation ^21, 39^, daylight hours ^25, 46^, solar radiation ^21, 31, 35^, and wind-speed ^27, 33, 35, 39^. One primary hypothesis of these studies was that these climatic factors and the dispersion of COVID-19 are correlated. In an attempt to examine the hypothesis, statistical and geospatial analysis was used as a key analytical tool ^25, 31, 39, 44^.

Studies analyzing the effects of temperature, humidity, and solar radiation found that an association exists between the transmission of the virus and humidity. These studies claimed that humidity is directly associated with the transmission ^31^, while temperature has no relation to the viruses diffusion ^35, 37^. However, another study refuted these findings and claimed both temperature and average daylight hours are influencing the spread of the virus ^25^. Other studies incorporated demographic variables as well as mobility and infections data with these meteorological factors to investigate how they are correlated with the COVID-19 outbreak ^35^. Instead of concentration mapping, they showed the spatial distribution and sensitivity of each factor in the study area map and asserted that population density and human mobility among the provinces are directly influencing the accelerated diffusion of COVID-19 cases.

Statistical and spatial analysis and modeling were used in other studies to identify the association between COVID-19 and climatic factors to understand the spatial distribution pattern of COVID-19 cases ^26, 44^ and spatio-temporal prediction of the pandemic for a different period ^36^. These studies concluded that without adequate and effective control measures, there is no evidence of humidity, and summer weather substantially limiting the pandemic growth ^26^. Additionally, Pearson's correlation, generalized additive model, and regression models were also utilized in different studies to understand the influence of climate on the virus transmission. These works widely used GIS for mapping the observed and predicted COVID cases ^30, 33^, mean temperature and humidity variation and correlation with COVID cases ^27^. None of these studies found any evidence of slower COVID-19 transmission with the change of temperature and humidity.

Different countries around the world have enforced different lockdown strategies since mid-January, even before WHO declared COVID-19 as a pandemic. As a result of these lockdowns, and lowered human and industrial activities, a substantial decline in air pollution was noticed ^23, 24, 28, 29, 34, 40, 41, 43^. Several studies had reported a significant reduction in NO _2_, CO _,_ and SO _2_ concentration in the environment ^23, 24, 28, 34, 40^. Studies reported significant improvement in AQI ^39^ and significant reductions in particulate matter (PM) concentrations ^41^. Similar studies at a global scale reported a significant decrease in NO _2 _concentration and minor decrease in CO concentration, and aerosol optical depth ^23^.

Other studies identified a global reduction of environmental pollution by up to 30%, human mobility by up to 90% ^24^, and vessel activities by up to 69% ^29^. A study using satellite images to compare the pollutants concentration before, during, and after the Chinese Spring Festival observed that the usual trend of NO _2_ and SO _2_ concentrations decrease before the festival and increase afterwards was not noticed in 2020 ^34^. ArcGIS was extensively used in several studies to quantify the association between AQI and the distribution of COVID-19 cases ^32, 33^. These studies claimed that air quality is a core driver of COVID-19 dispersion around the world, and the dispersion enhances in a temperature ranged from 10 to 20°C. ArcGIS was also used to perform sensitivity analysis, and for the calculation of Global Moran's I and LISA to analyze the impact of PM concentration in the air on the fatality rate in China ^45^. The study concluded that the fatality rate is positively correlated with the pollutant concentrations in the air.

***Socio-economic and cultural***. In response to the coronavirus pandemic, alongside the booming clinical and public health research, social scientists are also retooling existing studies, methodologies, and data to understand the people's behavior responding to the pandemic and its impact on socio-economic and cultural settings around the globe. A total of 12 articles were identified that emphasized on socio-economic and cultural aspects in COVID-19 and used GIS ^43– 54^. Researchers used gross domestic product (GDP), demographic and household compositions data, population density, and accessibility data ^46^. Several researchers utilized existing social vulnerability index data and applied geospatial tools and techniques to examine the spatial pattern of COVID-19 emergence across different socio-cultural settings ^47– 49^. Studies developed dot density and choropleth maps of COVID-19 cases and reported that population density diseases along with poverty and unemployment rates are the major indicators associated with a higher COVID-19 mortality rate ^49^.

Different studies reported association among spatial distribution of the socioeconomic variables and the temporal progression of the pandemic using a regression model based spatio-temporal analysis. They noticed that per capita GIS and public transit access is closely related to COVID-19 incidence ^46^. Using a similar analytical tool, another study estimated the association between virus infection and social vulnerability considering county-level socioeconomic data, demographic composition, disability and minority status, language, and housing and transportation database. The study concluded that the increase of COVID-19 cases are highly associated with minority status and language ^47^. To understand the association between racial inequality and COVID-19 mortality, Kim and Bostwick (2020) utilized principal component analysis (PCA) and hotspot analysis in ArcGIS. The study reported that African American communities are the ones with the highest COVID-19 related deaths in the USA ^48^. Other studies also used ArcGIS and regression analysis to investigate the spatial patterns of the COVID-19 in relation to socio-economic variables. This study used GIS to map the spatial aspects and disparities between metropolitan and nonmetropolitan communities and the regression analyses to test the hypotheses of positive correlations between COVID-19 incidence and mortality rates and socio-economic factors in the GIS ^50^.

Proximity and hotspot analysis in GIS has become a widely used geospatial analysis technique in the social research to understand the feasibility of social distancing ^51^, accessibility analysis of specific age group ^52, 53^. Gibson and Rush (2020) calculated the distance to each dwelling's nearest neighbors to identify units that are unable to practice social distancing effectively. Dryhurst *et al*. (2020) developed a global risk perception index (R.P.I.) using a linear regression model and used GIS to plot the mean risk perception of COVID-19 in 10 countries ^54^. Sarkar (2020) used ArcGIS to reclassify the administrative units of Bangladesh based on COVID-19 susceptibility using multicriteria analysis based pairwise comparison ^55^. Cavalcante and Abreu (2020) applied Moran’s I and LISA to identify the type and degree of spatial clustering and scatter plots of socio-economic indicators ^56^. Similarly, Exploratory Spatial Data Analysis (ESDA) technique was used to identify spatial relationships between the density of built heritage resources and Airbnb listings. Based on the calculation and mapping, they concluded that the distribution of Airbnb listings has a certain degree of spatial autocorrelation ^57^.

***Population health surveillance***. From the first case in Wuhan to the global pandemic, an enormous number of public health studies have been conducted to help the policymakers to understand how best to manage the current and future public health responses. A total of nine articles were identified that emphasized public health issues and used GIS ^50, 55– 62^. Multiple studies developed a multicriteria decision making index to assess the risk and resilience of the existing healthcare system. Requia *et al*. (2020) employed GIS techniques to construct a geodatabase comprising land use, income, population, health condition, number of hospital beds, and staff at the municipality scale. They predicted a deficit of 17 beds in Brazilian municipalities ^55^. Similarly, Jovanović *et al*. (2020) developed a global index comprising of 57 indicators using ArcGIS-based network analysis for hospital accessibility and resilience mapping ^58, 59^.

GIS was used to find out whether there is any association between orthopedic surgeons’ age and COVID-19 confirmed cases ^60^. The study reported a spatial relationship between the number of cases and number of surgeons in a state. In a similar study, Ruthberg *et al*. (2020) studied the potential risk of otolaryngologists above age 60 due to COVID-19, where they used a heat map to show the state-wise potential risk zone using QGIS. The study indicated that New York, New Jersey, Massachusetts, and Michigan were the riskiest zone according to the ratio of the number of confirmed cases to the number of total ENT's over 60 ^61^. Kuupiel *et al*. (2020) calculated the distance from the primary healthcare (PHC) clinic as well as to the nearest health facility in ArcGIS to measure geographical accessibility (in terms of distance and time) to COVID-19 specialized hospital facilities in Ghana. The analysis reported that the current mean travel time (more than an hour) and distance (more than 35 kilometers) to PHC is significantly higher than the globally accepted standards ^62^. Similar accessibility analysis was done in Australia using proximity analysis and hotspot analysis to measure travel time to the closest hospital facility for aged population resulted in a similar outcome as well ^53^.

Ahmadi *et al*. (2020) developed an epidemic prediction model for spatial-temporal analysis to predict and estimate the number of patients and deaths at the end of pandemic (infected, cured, and dead cases). The study predicted that approximately 7900 and 4620 deaths would occur in Iran from May 13 to June 1, 2020, respectively, and then the curve will flatten ^63^. To analyze the epidemiology of COVID-19, studies also utilized georeferencing. These studies first geocoded all COVID-19 cases and then joined them to the county layers by administrative codes in ArcGIS and afterwards, applied LISA. This study reported that the spatial clustering is not random and shows significant spatial heterogeneity in China ^64^. A wastewater-based epidemiology (WBE) tool was proposed as a surveillance tool to monitor the COVID-19 pandemic ^65^. The study used multiple variables to run the GIS-based hydraulic model and network analysis using the SWMM modeling environment and ArcGIS. The result effectively served as a justification to use WBE as a rapid and efficient tool to track COVID-19, which the authors claimed could be used with clinical testing to save billions of dollars ^65^.

***Spatial pattern analysis of COVID-19***. A total of nine articles were identified that emphasized on identifying the spatial transmission pattern analysis of COVID-19 ^14, 16, 19, 62– 67^. So far, only one study had used geospatial analysis to identify Spatio-temporal clusters and prediction modeling for COVID-19 transmission. They utilized the Poisson probability distribution model, Kernel density analysis, and space-time scanning analysis to identify high-risk Spatio-temporal clusters for transmission of COVID-19 in Brazil and detected active Spatio-temporal clusters comprising six municipalities in the south-central region of Brazil ^66^. Only two studies so far studied how travel restrictions may have limited the epidemic trajectory. One of them developed a global model based on internationally reported cases and mobility data, to project the impact of travel limitations on the national and international spread of the epidemic and revealed that Wuhan travel ban only hindered the overall epidemic trajectory by 3 to 5 days in other cities of China but had a significant influence on the international scale dispersion ^22^. Studies also estimated the probability of COVID-19 cases transportation before January 23 among 369 cities in China and reported that 130 cities in China have more than 50% chance of having a COVID-19 case imported from Wuhan ^67^.

In a global study to identify geographic risks of COVID-19 transmission using online Rasch Modeling Algorithm, the authors reported that Iran, South Korea, Italy, Germany, Spain, China (Hubei), and France, are the top countries with higher outbreak potential respectively ^68^. Other studies utilized accessibility analysis techniques to assess the spatial diffusion of COVID-19, using GIS-based origin, destinations, and frequencies mapping of public transportation from Wuhan. The study claimed that increase of COVID-19 cases have a direct relationship with the frequency of public transport from Wuhan ^69^. Other studies utilized time-series analysis and plots to portray the spatial and temporal variation of COVID-19 cases in China and to elucidate the role of case importation in transmission in cities across China using spatial analysis ^70^.

One other global study used GIS to visualize the worldwide distribution of air transport passengers from Wuhan and infected traveler’s ratio around the world ^19^. Geo-referencing of confirmed cases also played an interesting role in the spatial pattern analysis studies. These studies measured and identified the regions that have a high risk of transmission at an early stage ^71^. Similarly, a generalized linear regression model was used to analyze the spread and control of COVID-19 cases using case reports, and human movement data. This study utilized spatial distribution mapping of the travel movements from Wuhan to each province and modeled the spatial dispersal pattern of COVID-19 trajectories with or without Wuhan travel ban. They found that Wuhan shutdown delayed arrival of COVID-19 in other cities by around three days ^18^.

***Computer-aided spatial and statistical analysis and modeling***. A substantial number of studies were found to apply computer-aided spatial and statistical analysis and modeling techniques in analyzing distinct aspects of COVID-19. A total of 16 articles were identified that emphasized on computer-aided spatial and statistical analysis and modeling in COVID-19 ^69– 84^. Most of these works were focused on analyzing the spatial distribution pattern of COVID-19 cases using the confirmed cases data ^72– 78^, or news reports of COVID-19 cases as proxy data ^79^. Most of the studies were focused on examining the spatial distribution and relationship between COVID-19 cases, deaths, and locations. Adekunle *et al*. (2020) examined the same relationship. They found a positive statistically significant relationship among spatial clusters, confirmed cases and potential deaths ^72^. Another similar study in Hubei, China, demonstrated that the high-low cluster had no high-value incidence cluster where local Moran's I indicated that Hubei was the only province with High-Low aggregation ^74^. Moran's I was applied in analyzing the spatial and temporal distribution of cases in Hubei province by other studies as well ^77^.

Multiple studies examined the spatial and temporal distribution and modeled the trend of COVID-19 cases growth in ArcGIS and found that the highest risk place was those that had a high population inflow from Wuhan and Hubei province ^75, 76^. Spatial modeling using Poisson space-time scan statistics to produce cluster mapping attempts also led to the proposal of first rapid surveillance to monitor the spread of COVID-19 ^73^. Miller *et al*. (2020) demonstrated the worldwide spatial distribution of COVID-19 cases using the heat map technique in GIS The map reported that China, Italy, Iran, and Spain were the highest affected countries till March 17, 2020, which was also visible by the JHU and WHO reported data ^78^. Spatial panel data model used by Guliyev (2020) showed that the rate of deaths had a significant positive effect where the recovery rate had a negative with the confirmed COVID-19 cases ^80^. Irvine *et al*. (2020) used a SEIR model to estimate the transmission rate within Immigration and Customs Enforcement detention facilities in the GIS and the impacts on the I.C.U. capacity ^81^.

Bai (2020) used two different models, including SEIRD model and Agent-Based Model (A.B.M.) to simulate the COVID-19 spread. They found that A.B.M. could be more effective and it also could be a useful tool to figure out new effective strategies ^82^. In a similar work, Mollalo *et al*. (2020) compared five different models to develop a spatial model of COVID-19 incidence rate considering 35 variables using geospatial software’s. The results showed that MGWR could be a better model as it was able explain 68.1% of the total variation of COVID-19 incidences in the GIS ^83^. In a follow-up paper, the same authors used ANN to model the incidence rate of COVID-19 and used Moran's I index to create incidence hotspots. Out of included 57 variables, 10 variables found statistically significant in explaining the result ^84^. A similar global scale study revealed that age and population density have a statistically significant relationship with the spatial distribution pattern of COVID-19 cases ^85^.

Using Maxent based Ecological Niche Model, Ren *et al*. (2020) developed a potential risk zone map of China where population, public transportation demands, medical resources demands were used as explanatory variables. They suggested using this as an early forecasting model to predict the risk zone in China's other megacities ^86^. Kanga *et al*. (2020), on the other hand, provided a useful recommendation to local authorities in India by using proximity-based hot spot analysis to map the risk zones with relevant preventative measures to mitigate the COVID-19 crisis ^87^. Several other works were focused on suitability mapping with a focus to find a suitable location for the quarantine zone in Surat, India, using ArcGIS. The study revealed that the suitability analysis could help to control the spread as a prevention measure.

## Data mining and COVID-19

We grouped the application of big data, social media data mining, and contact tracing through geospatial technologies together. Although very few studies attempted to incorporate those techniques in COVID-19 research, a total of eight articles were identified that emphasized on data mining, big data, and social media data use in COVID-19 ^62, 85– 91^. Data mining using unsupervised machine learning models were utilized in a study to analyze the twitter data and it reported tweets related to symptoms of users is associated with COVID-19 testing accessibility ^66^. Studies also used nighttime light (NTL) data and AQI data to analyze the spatial and temporal pattern of COVID-19 and how that impacted human activities. The observation demonstrated that the NTL brightness and AQI value were much lower during the quarantine period in Mainland China ^88^. Similarly, radar data was used to detect traffic patterns where the findings reported that the number of heavy vehicles movement in the region changed significantly after the COVID outbreak ^89^.

Several studies used mobile sensor data or geodata for contact tracing as a surveillance strategy to monitor COVID-19 ^90– 92^. Wang *et al*. (2020) developed a Geo-AI based mini program within an instant messaging app (WeChat) to trace close contacts of all confirmed patients. The results showed that the program could analyze real-time data to trace the contacts, and those data could be used with other datasets to find out more useful information to reduce COVID-19 spread. Similarly, other studies also attempted to develop a smart contact tracing app using big data analytics ^90^ or using a mobile sensor-based contact tracing system to minimize the spread of COVID-19 ^91^. Using social media posts, Huang *et al*. (2020) examined the attributes of both suspected and confirmed COVID-19 cases who contacted with the symptoms. They used SPSS and ArcGIS for descriptive statistical analysis and spatial analysis, where they found that most of the patients seeking help were above 65 years old from Wuhan ^93^.

## Discussion

To the best of our knowledge, this is one of the first systematic reviews of the application of GIS and other geospatial technologies in COVID-19 related research. Our work does not only provide an overview of how GIS was used so far but also provides pointers on how GIS could be more efficiently used in COVID-19-related works and other public health issues in the coming days. The application of GIS technologies and spatial analysis has substantially influenced the understanding of COVID-19, not only for the scientific community but also for the policymakers, and for the public in building a long term response to the ongoing pandemic ^94^. Initially, spatial analysis techniques were used as part of predictive modeling to predicts the growth of COVID-19 cases ^63^ and to model the spatio-temporal variation of confirmed incidences ^74^. With the increasing availability of COVID-19 data, a significant number of studies started to analyze the spatial transmission pattern and spread of the virus from Wuhan to other cities in China and the rest of the world ^17, 18, 20, 22, 26, 67, 71, 89, 95^. Most of these early applications of GIS and spatial analysis were more focused on visualizing the COVID-19 confirmed cases as well as the distribution of cases among administrative units and countries. However, as time goes on and more data became available, more complex GIS tools come into play. Studies not only used GIS for analyzing different environmental aspects; they also used different earth observation data acquired by the European Space Agency (ESA) and NASA ^23, 24, 28, 34^.

During the early days, one of the biggest discussions among the researchers was regarding the ability of meteorological factors to limit the spread of coronavirus. With the declaration of the pandemic and subsequent lockdown globally in early March, several studies used GIS and remotely sensed images to analyze the impacts of this lockdown on the environment, air quality, and other particulate matters ^18, 23, 26^. Several studies evaluated that relationship with the help of spatial analytical tools, and most of these studies could not reach a valid conclusion where they could claim temperature or other climatic factors do limit the spread of the virus ^23, 24, 27, 30^. Later application of GIS does not restrict itself in just visualizing. Instead, it was more used to spatial autocorrelation and clustering analysis, hotspot analysis, and suitability analysis to see whether any association exists among social and economic groups, any specific location or social group, and COVID-19 infection rate ^55, 56, 59^. With the increasing confirmed cases around the world, different social-science studies analyzed the association among COVID-19 infection rate and social vulnerability, racial inequality, risk perception, resiliency, and settlement quality issues ^44, 45, 47^. These studies also utilized GIS to identify any spatial patterns and autocorrelation with COVID-19.

We reviewed how GIS and spatial analysis techniques were used in the past COVID-19 related studies. We found that most of the included studies used GIS for visualizing the spatial distribution and pattern of COVID-19 spread, cluster analysis to identify the accumulation of cases, hot spot analysis to find out any outbreak, proximity analysis to evaluate the accessibility to the primary health care facilities. However, with time, studies focused on different models to predict or simulate various aspects of COVID-19 using geospatial techniques that were published as well. On that point of interest, GIS-based Maxent model, spatial data panel model, SEIR model, Agent-Based Model, GWR, MGWR, ANN were used in different studies ^80– 84, 86^. However, we did not find any studies that used spatially explicit modeling to identify and predict the location of any potential outbreak in the future. One of the few positive aspects of COVID-19-related studies is the publicly available data, and the same was noticed in the review as well. We found that more than half of the studies used data from some form of government database followed by WHO database (n=12), different websites (n=12), JHU dashboard and Worldometers (n=9), Social media data (n=8), satellite images (n=7), primary survey (n=3) and mobile phone data (n=3).

Though COVID-19 related data is mostly publicly available, some studies reported data unavailability issues, especially in developing or less developed countries ^32, 49, 84^. The major challenge of global or regional studies is the possibility of an under-reported number of confirmed cases, especially in low-income regions, because of the low detection coverage of COVID-19, which may skew the result. Most of the global or regional studies cannot incorporate the controlling measures imposed by different governments, which has significant impacts on the spread and infection incidences of COVID-19 cases. No consideration of government control measures and low testing issues is also a substantial limitation for modeling and prediction focused research which creates a biased result. Therefore, future studies should emphasize considering government control measures and policies in their modeling. Although contract tracing and data mining research has been proven to be useful in analyzing and forecasting the spatial pattern of COVID dispersion, no trace of any studies were found outside of China and the USA. That might be due to data unavailability and technological issues. Contract tracing in China was possible due to its government-backed app that gathers a user's information, including name, ID number, and health information and movement data. Two studies were conducted in China and Taiwan for smart contract tracing using mobile sensor data ^90, 92^. Low or middle-income countries can adopt a GIS-based volunteered surveillance approach where peoples will share their information voluntarily to tackle the pandemic. So far, GIS has not been used much to track the transmission pattern and to predict the transmission. That is something that can be done at a global level to leverage GIS to predict not only the confirmed case numbers but also specific locations where the outbreak would happen with higher statistical precision ^96^.

The findings of this review have profound implications for contemporary and future multidisciplinary scientific research, policymaking, and practice. The diverse use of GIS technologies in different overarching thematic areas of scientific research highlights the potential of incorporating methodological perspectives for solving complex research questions. This evolution is consistent with the emerging perspective that “one size does not fit all” and each unique scenario may require conceptual and empirical inputs from different disciplines for achieving a higher precision on research outcomes. Nonetheless, an increasing trend of integrating GIS technologies in studies that emphasized on multiple research objectives show how such technologies are being a part of the entire work rather than the only approach used in those research efforts. Thus, the use of GIS may improve other methodological measures and increase the scope of scientific exploration on a topic of interest.

The existing evidence highlights the use of GIS and other geospatial techniques for addressing research question; however, little evidence exists on how geospatial can be used for delivering digital interventions for individuals or target populations. Perhaps such technological innovations would take much time to appear, but precision sciences and their applications on personalizing user level platforms may bring such technologies more closer to everyday practice. Moreover, a wide range of data sources used in different studies included in this review provide meaningful insights on how data from multiple can be harmonized and utilized in addressing population-based problems. Furthermore, integrating GIS in COVID-19 related research may enable real-time decision-making for preventing public health crises and deploying resources whenever required. A major lesson from existing studies is to developing local and global disaster preparedness plans that may enable policymakers and practitioners to leverage GIS-based advanced data analytics for mitigating large scale public health emergencies. More implementation research is needed to assess the scope of such multipronged yet coordinated response systems that may emerge in the post-pandemic world. Such initiatives may require strengthening technological capacities in low and middle-income countries that share a major proportion of global health problems, yet have limited resources to address the same ^97, 98^.

Despite notable strengths, this systematic review has several limitations that should be acknowledged and addressed in future research. First, the selection of databases and keywords could have excluded some studies that were indexed in other databases or used non-specified keywords, which were beyond the scope of this review. Second, we focused on peer-reviewed publications and did not cover preprints that did not undergo peer-review; therefore, those studies are also excluded from this review, which may provide further insights on the evidence landscape. Third, the existing literature shows a high heterogeneity in the methods, data inputs, and research outcomes leading to a narrative synthesis. Fourth, we did not assess the quality of the studies and the risk of bias within and between the studies. We recommend that future evidence syntheses on specific GIS-related topics should assess the risk of bias among the scientific literature in those topics. Prospective evidence-based reviews may also consider the quantitative synthesis of homogenous studies on specific themes. This systematic review provides an inclusive and extensive synthesis of multidisciplinary research using GIS during COVID-19, which may inform future primary studies and advanced syntheses addressing the current limitations and improving the knowledge base in this domain.

## Conclusion

This systematic review evaluated the current literature on the use of GIS and geospatial analyses in the context of COVID-19 pandemic and explored the scope of integrating such techniques in the current research efforts as well as future research and practice. In the era of digital revolution, a growing need for exchanging technological advancements across scientific disciplines is widely acknowledged. The use of GIS and related technologies in COVID-19 pandemic examplifies such integrations and provide scholarly perspectives on how complex societal and global issues can be understood using the existing tools. Moreover, such applications necessitate revisiting the current strengths and weaknesses of curating evidence across contexts. It is essential to strengthening institutional capacities to leverage GIS-related technologies in multipronged research and development that empower research communities to work together in this pandemic. Last but not least, future technological innovations should be grounded on the lessons learned during this pandemic to make such technologies readily available for facilitating robust research and decision-making that may improve population-level outcomes globally.

## Data availability

### Underlying data

All data underlying the results are available as part of the article and no additional source data are required.

### Extended data

Figshare: Application of geospatial techniques in COVID-19-related studies. https://doi.org/10.6084/m9.figshare.13229147.v2 ^16^.

This project contains a summary of the articles idenitifed in this study. This includes a detailed breakdown of what methods used in the reviewed article, along with the data type, spatial analysis tool/techniques and the findings from the analysis.

### Reporting guidelines

Open Science Framework: PRISMA checklist for ‘Applications of GIS and geospatial analyses in COVID-19 research: A systematic review’. https://doi.org/10.17605/OSF.IO/ZGMP8 ^99^.

Extended data are available under the terms of the Creative Commons Attribution 4.0 International license (CC-BY 4.0); the compelted PRISMA checklist is available under the terms of the Creative Commons Zero "No rights reserved" data waiver (CC0 1.0 Public domain dedication).
