# Supplementary material for: Applications of GIS and geospatial analyses in COVID-19 research: A systematic review
Source: F1000Res. 2022 Jan 28;9:1379. Originally published 2020 Nov 27. [Version 2] doi: 10.12688/f1000research.27544.2 (PMC8822139; doi:10.12688/f1000research.27544.2)
Supplement: Peer review history

# Peer review: Reviewer response for version 2

**DOI:** 10.5256/f1000research.120587.r121498

**Published:** 2022-02-07

**Authors:** Orhun Aydin

**Affiliations:** 1 Spatial Sciences Institute, University of Southern California, Los Angeles, USA

I do not have any further revisions.

Are the rationale for, and objectives of, the Systematic Review clearly stated?

Yes

Is the statistical analysis and its interpretation appropriate?

Not applicable

Are sufficient details of the methods and analysis provided to allow replication by others?

Yes

Are the conclusions drawn adequately supported by the results presented in the review?

Yes

Reviewer Expertise:

NA

I confirm that I have read this submission and believe that I have an appropriate level of expertise to confirm that it is of an acceptable scientific standard.

# Peer review: Reviewer response for version 2

**DOI:** 10.5256/f1000research.120587.r121497

**Published:** 2022-01-31

**Authors:** Hamid Mukhtar

**Affiliations:** 1 Department of Computer Science, College of Computers and Information Technology, Taif University, Taif, Saudi Arabia

I have seen the authors' reply and they have addressed the limitations.

Are the rationale for, and objectives of, the Systematic Review clearly stated?

Yes

Is the statistical analysis and its interpretation appropriate?

Partly

Are sufficient details of the methods and analysis provided to allow replication by others?

Yes

Are the conclusions drawn adequately supported by the results presented in the review?

Yes

Reviewer Expertise:

computer science, information visualization, data analytics, machine learning, deep learning

I confirm that I have read this submission and believe that I have an appropriate level of expertise to confirm that it is of an acceptable scientific standard.

# Peer review: Reviewer response for version 1

**DOI:** 10.5256/f1000research.30442.r97367

**Published:** 2021-12-16

**Authors:** Orhun Aydin

**Affiliations:** 1 Spatial Sciences Institute, University of Southern California, Los Angeles, USA

The sentence “Although GIS has substantial potential in planning to slow down the spread, surveillance, contact tracing, and identify the trends and hotspots of breakdowns, it was not employed as much as it could have been.” Can be expanded. This sentence can also include another vital role that GIS played, putting data on a map to inform the public. WebGIS was particularly crucial during the pandemic for keeping the public informed of the spread of COVID-19.

 In the introduction the sentence “Although the first reported case was in China, and it was the epicenter of the pandemic, the virus has mutated and changed transmission pattern several times since then.” needs a reference.

 The sentence "Besides RStudio, QGIS (n=4) is another open source software platform that was highly employed by the researchers." RStudio is a software for programming whereas QGIS is a software platform for GIS". I think QGIS and ArcGIS would fare better if they were in the same sentence.

 Paragraph starting with "ESRI ArcGIS is the most used platform" has to mention Python. Most labs forecasting the spread of the virus IHME, CHIME to name a few, provided Python libraries and they were extensively used by decision-makers.

 SIGSPATIAL had two conferences on COVID. Below are references from the main paper and one of our contributions. Note that in the statement "SEIR model, Agent-Based Model, GWR, MGWR, ANN were used in different studies" you will see in this workshop that there were studies that used GIS and SEIR, SIR-type epidemiological models.

 When data challenges are discussed an important point to inform the reader on would be confidentiality.

Are the rationale for, and objectives of, the Systematic Review clearly stated?

Yes

Is the statistical analysis and its interpretation appropriate?

Not applicable

Are sufficient details of the methods and analysis provided to allow replication by others?

Yes

Are the conclusions drawn adequately supported by the results presented in the review?

Yes

Reviewer Expertise:

Spatial statistics, GIS, space-time statistics, Earth science, human-environment interaction, geostatistics.

I confirm that I have read this submission and believe that I have an appropriate level of expertise to confirm that it is of an acceptable scientific standard, however I have significant reservations, as outlined above.

## References

[1] : The 1st ACM SIGSPATIAL International Workshop on Modeling and Understanding the Spread of COVID-19. *SIGSPATIAL Special* .2021;12(3) : 10.1145/3447994.3448007 35-40 10.1145/3447994.3448007

[2] : Sensitivity analysis for covid-19 epidemiological models within a geographic framework. *Proceedings of the 1st ACM SIGSPATIAL International Workshop on Modeling and Understanding the Spread of COVID-19* .2020; 10.1145/3423459.3430755 11-14 10.1145/3423459.3430755

# Response

**Published:** 2022-01-23

**Authors:** Rakibul Ahasan

**Affiliations:** Texas A&M University, College Station, USA

We thank you for your comments on our manuscript. We believe addressing the comments helped to improve the quality of our work. Please find the point-by-point responses and revision details below-

- The sentence “Although GIS has substantial potential in planning to slow down the spread, surveillance, contact tracing, and identify the trends and hotspots of breakdowns, it was not employed as much as it could have been.” Can be expanded. This sentence can also include another vital role that GIS played, putting data on a map to inform the public. WebGIS was particularly crucial during the pandemic for keeping the public informed of the spread of COVID-19.

GIS maps, especially web maps and WebGIS-based dashboards, played an important role in informing the public regarding the spread of the virus, especially during the early days of the pandemic. Therefore, in addition to our previous statement, we have included *Geospatial techniques, especially WebGIS, which have even been widely used in putting the data into a map and were critical to informing the public regarding the spread of the virus especially during the early days of the pandemic*.

- In the introduction the sentence “Although the first reported case was in China, and it was the epicenter of the pandemic, the virus has mutated and changed transmission pattern several times since then.” needs a reference.

We included references to support our statement regarding the first reported case. We used one of the most evidence-based, peer-reviewed works we could find in PubMed ( **Alam, 2020**). For the virus mutation and transmission pattern, we used a reference that discussed the chronological mutation of the virus- from the original variant to the most recent Omicron variant ( **Kannan et al., 2022**).

 Allam Z. (2020). The First 50 days of COVID-19: A Detailed Chronological Timeline and Extensive Review of Literature Documenting the Pandemic. *Surveying the Covid-19 Pandemic and its Implications*, 1–7. https://doi.org/10.1016/B978-0-12-824313-8.00001-2

 Kannan, S. R., Spratt, A. N., Sharma, K., Chand, H. S., Byrareddy, S. N., & Singh, K. (2022). Omicron SARS-CoV-2 variant: Unique features and their impact on pre-existing antibodies. *Journal of autoimmunity*, *126*, 102779. https://doi.org/10.1016/j.jaut.2021.102779

- The sentence "Besides RStudio, QGIS (n=4) is another open-source software platform that was highly employed by the researchers." RStudio is a software for programming whereas QGIS is a software platform for GIS". I think QGIS and ArcGIS would fare better if they were in the same sentence.

We initially grouped QGIS with RStudio as both are free and open-source software. However, we agree based on the functionality, QGIS better fits with ESRI ArcGIS. Therefore, we changed the sentences accordingly.

- Paragraph starting with "ESRI ArcGIS is the most used platform" has to mention Python. Most labs forecasting the spread of the virus IHME, CHIME to name a few, provided Python libraries and they were extensively used by decision-makers. SIGSPATIAL had two conferences on COVID. Below are references from the main paper and one of our contributions. Note that in the statement "SEIR model, Agent-Based Model, GWR, MGWR, ANN were used in different studies" you will see in this workshop that there were studies that used GIS and SEIR, SIR-type epidemiological models.

As the latest date of our review was June 28, 2020, papers published after that date were out of our review's scope. However, we strongly agree it is important to discuss these epidemiological models that were used to forecast the spread of the virus within a geographic context. Therefore, we revised the discussion section to include the use of epidemiological models. At the same time, we believe future systematic reviews would include these works that were published later, which was beyond the scope of our review.

- When data challenges are discussed an important point to inform the reader on would be confidentiality.

This is an important point. Data confidentiality has been widely discussed not only in public health research but also in other domains as well. Even geospatial techniques using satellite images and remote sensing has been facing backlashes over data confidentiality issues. We call for robust data management and sharing practices ensuring confidentiality and ethical use of data. 

 We express gratitude to the reviewer for their time and valuable comments and hope this updated, revised version of our work is more comprehensive and satisfactory.

# Peer review: Reviewer response for version 1

**DOI:** 10.5256/f1000research.30442.r95380

**Published:** 2021-10-25

**Authors:** Hamid Mukhtar

**Affiliations:** 1 Department of Computer Science, College of Computers and Information Technology, Taif University, Taif, Saudi Arabia

This article reviews existing approaches in the use of geographical information systems (GIS) for COVID-19 containment, contact tracing, surveillance and hotspots identification. Further, it suggests how GIS can be used by the scientific community and policy-makers for informed decision-making.

 The article uses the PRISMA approach for systematic analysis of the application of GIS in COVID-19. The approach is explained clearly. The outcome of the study with various dimensions has been discussed. The limitations have also been discussed.

 The MEDLINE and Web of Science repositories were used as data sources. My concern is that there are GIS specific journals and conferences organized by organizations like IEEE and ACM; have authors tried to explore those venues for better results? Why not? Apparently, GIS in COVID-19 should be searchable in computing and engineering databases rather than medical databases.

 ​On the positive side, the authors have created thematic groups from the review, which is useful for readers.

 I suggest that the authors add a summary table that can provide the considered dimensions, attributes, considerations, etc. Figure 2 may be reconsidered without the world map.

 The quality of figure 1 is not very good, should be improved.

Are the rationale for, and objectives of, the Systematic Review clearly stated?

Yes

Is the statistical analysis and its interpretation appropriate?

Partly

Are sufficient details of the methods and analysis provided to allow replication by others?

Yes

Are the conclusions drawn adequately supported by the results presented in the review?

Yes

Reviewer Expertise:

computer science, information visualization, data analytics, machine learning, deep learning

I confirm that I have read this submission and believe that I have an appropriate level of expertise to confirm that it is of an acceptable scientific standard.
